# Contraception in Women With Medical Comorbidities: Clinical Risk Stratification and Individualized Therapeutic Strategies

**DOI:** 10.7759/cureus.105102

**Published:** 2026-03-12

**Authors:** Laura C Castro Rivero, Eimy Zúñiga Gómez, Pablo R Durán Monge

**Affiliations:** 1 Gynecology, Hospital San Juan de Dios, San José, CRI; 2 Critical Care, Soporte Asistido Aéreo y Terrestre, San José, CRI

**Keywords:** autoimmune disease, chronic illness, drug interactions, eligibility criteria, maternal morbidity, metabolic dysfunction

## Abstract

Women of reproductive age increasingly present with chronic medical conditions that significantly influence contraceptive safety and effectiveness. Hormonal contraceptives, particularly estrogen-containing formulations, exert systemic effects on coagulation pathways, vascular tone, blood pressure regulation, and metabolic function. These mechanisms contribute to an increased risk of venous thromboembolism, hypertension, and cardiovascular events in susceptible populations, especially in women with obesity, diabetes mellitus, dyslipidemia, autoimmune disorders, or pre-existing cardiovascular disease. Progestin-only methods generally demonstrate a lower thrombotic risk profile; however, their metabolic and systemic effects vary depending on the specific formulation. Long-acting reversible contraceptives, including levonorgestrel-releasing and copper intrauterine devices, offer highly effective options with minimal systemic impact and are frequently recommended for women with contraindications to estrogen.

Comorbid conditions such as systemic lupus erythematosus, antiphospholipid syndrome, chronic liver disease, chronic kidney disease, and hormone-sensitive malignancies further complicate contraceptive decision-making. In these settings, individualized risk assessment must consider organ-specific vulnerability, disease activity, and potential drug-drug interactions, particularly with enzyme-inducing medications or immunosuppressive therapies. Evidence-based eligibility classification systems provide structured guidance for identifying absolute and relative contraindications and support consistent clinical practice.

Effective contraceptive care in medically complex patients requires multidisciplinary collaboration, shared decision-making, and reproductive life planning. Comprehensive counseling, regular monitoring, and timely method adjustment are essential to reduce unintended pregnancies, prevent disease exacerbation, and minimize maternal morbidity. Ultimately, tailored contraceptive strategies that integrate pathophysiological understanding with patient-centered care are critical to optimizing reproductive and overall health outcomes in women with comorbidities.

## Introduction and background

Chronic medical conditions are highly prevalent among women of reproductive age. Current evidence indicates that approximately one in four women enters pregnancy with a pre-existing condition such as diabetes mellitus, hypertension, or psychiatric disorders [1]. The increasing burden of multimorbidity further complicates reproductive health management, as the coexistence of multiple chronic conditions significantly increases the risk of severe maternal morbidity and postpartum readmission [2]. In this context, women living with chronic illnesses face a higher likelihood of unintended pregnancy, which is frequently associated with adverse maternal and perinatal outcomes. This intersection between chronic disease and reproductive health highlights the importance of proactive and individualized contraceptive strategies [3].

Unintended pregnancy in women with chronic conditions reduces opportunities for timely preconception care and counseling, both of which are essential for optimizing disease control prior to conception. The absence of adequate pregnancy planning may exacerbate underlying medical conditions during gestation and increase the risk of maternal and fetal complications. For example, women with sickle cell disease report high rates of unintended pregnancy alongside limited knowledge of effective contraceptive options, illustrating persistent gaps in reproductive health education and access to appropriate counseling [4]. Furthermore, the presence of multiple chronic conditions has been directly associated with a higher risk of severe maternal morbidity and mortality, reinforcing the need for effective contraceptive strategies aimed at preventing unintended pregnancies [1].

Maternal mortality remains disproportionately elevated among women with chronic illnesses. In particular, non-Hispanic Black and Native American women experience mortality rates two to three times higher than those observed among White women [5]. Certain medical conditions, including systemic lupus erythematosus and antiphospholipid syndrome, are associated with particularly high risks of maternal and fetal complications, thereby requiring careful clinical management and meticulous reproductive planning [6]. At the global level, maternal disorders continue to represent a major public health burden, with maternal hemorrhage identified as the leading cause of death among women of reproductive age, further underscoring the importance of preventive strategies [7].

Given these risks, appropriate contraceptive selection represents a critical component of care for women with chronic medical conditions. Tailored contraceptive strategies can help prevent high-risk pregnancies and improve overall maternal health outcomes [8]. Personalized contraceptive counseling, including the use of decision-support tools, has been shown to enhance informed decision-making and strengthen reproductive autonomy in this population [3]. However, disparities in contraceptive counseling persist, particularly among women with cardiovascular disease, who frequently receive inconsistent recommendations and may consequently rely on less effective contraceptive methods that do not adequately reflect their clinical needs [9].

The objective of this review is to synthesize current evidence regarding the impact of chronic medical conditions on reproductive health outcomes and to examine the role of individualized contraceptive strategies in reducing unintended pregnancy, severe maternal morbidity, and mortality among women with comorbidities, with particular emphasis on risk-adapted clinical decision-making and patient-centered counseling.

## Review

Methodology

This study was designed as a narrative review with qualitative evidence synthesis to summarize contemporary knowledge on contraceptive use in women with medical comorbidities. Narrative reviews allow integration of clinical evidence, pathophysiological mechanisms, and guideline recommendations relevant to complex clinical scenarios. In this review, the synthesis was organized according to major categories of chronic conditions, including cardiovascular, metabolic, autoimmune, neurological, hepatic, renal, and oncologic disorders. These categories were analyzed in relation to clinically relevant outcomes such as unintended pregnancy, severe maternal morbidity, thromboembolic risk, disease exacerbation, and contraceptive safety profiles. The methodological approach aimed to integrate pathophysiological insights with clinical applicability in high-risk populations of reproductive age.

A structured literature search was conducted in PubMed, Scopus, and ScienceDirect to identify relevant peer-reviewed publications. The search included studies published in English or Spanish between January 2020 and December 2025 in order to capture recent developments in contraceptive safety, updated medical eligibility criteria, and emerging evidence regarding reproductive health in women with chronic diseases. The search strategy combined Medical Subject Headings and free-text terms using Boolean operators, including (“contraception” OR “hormonal contraception” OR “long-acting reversible contraception”) AND (“chronic disease” OR “comorbidities” OR “cardiovascular disease” OR “diabetes” OR “autoimmune disease”) AND (“maternal morbidity” OR “unintended pregnancy” OR “thromboembolism” OR “risk stratification”) AND (“medical eligibility criteria” OR “contraceptive counseling”). The initial search identified 214 records. After removal of duplicates and title-abstract screening, 102 articles underwent full-text evaluation, and 43 studies were included in the final qualitative synthesis. Study selection and evaluation were conducted independently by two authors, with discrepancies resolved through discussion and consensus.

Eligible sources included randomized controlled trials, large cohort studies, population-based observational studies, systematic reviews, meta-analyses, and international clinical guidelines addressing contraceptive use in women with chronic medical conditions. Studies were selected based on their relevance to contraceptive safety, risk stratification, or clinical management in medically complex populations. Priority was given to studies reporting clinically meaningful outcomes, clearly defined safety endpoints, adequate follow-up duration, and robust methodological design. Studies limited to small uncontrolled case series, non-peer-reviewed reports, or investigations involving populations without clearly defined comorbid conditions were excluded.

Although formal risk-of-bias tools commonly used in systematic reviews were not applied, the methodological quality of the included studies was evaluated narratively. This qualitative appraisal considered study design, potential risk of bias, sample size, follow-up duration, clarity of outcome definitions, and external validity. When conflicting findings were identified, greater interpretative weight was given to higher-level evidence and guideline-supported recommendations. Because of the narrative nature of this review, no pooled statistical analysis, meta-analysis, or meta-regression was performed, and quantitative outcomes reported in the manuscript originate from the cited primary studies.

Artificial intelligence-based tools were used solely for structural organization and linguistic refinement of the manuscript. All scientific interpretation, literature selection, and final conclusions were determined exclusively by the authors to ensure academic rigor and integrity.

Pathophysiological considerations and risk stratification

Estrogen-containing contraceptives exert significant effects on coagulation, blood pressure, and vascular tone, which are central to understanding their cardiovascular risk profile. Estrogen increases the risk of venous thromboembolism by altering clotting factor synthesis and promoting resistance to activated protein C, a key regulator of thrombin generation [10,11]. In addition to its procoagulant influence, estrogen modulates the renin-angiotensin-aldosterone system and affects the balance between vasodilatory and vasoconstrictive mediators, mechanisms that may contribute to elevations in blood pressure and the development or worsening of hypertension [12]. These physiological effects underscore the importance of careful cardiovascular evaluation when prescribing estrogen-containing methods. Progestins, although often considered safer than estrogen with respect to thrombotic risk, demonstrate heterogeneous metabolic and cardiovascular effects depending on the specific formulation. For example, depot medroxyprogesterone acetate has been associated with an increased risk of venous thromboembolism, whereas levonorgestrel-based systems have not shown the same elevation in risk [13]. Beyond thrombosis, progestins can influence lipid metabolism and insulin sensitivity, thereby contributing to cardiometabolic risk factors such as dyslipidemia and diabetes [14]. 

Hormonal contraceptives also interact with inflammatory and autoimmune pathways. Their immunomodulatory effects may exacerbate conditions such as systemic lupus erythematosus, which is itself associated with an increased thrombotic risk. Estrogen’s role in immune regulation can influence autoimmune disease activity, necessitating vigilant monitoring in affected women. Accordingly, the selection of contraceptive methods in this population must account not only for thrombotic risk but also for potential impacts on disease stability [13].

Thromboembolic risk varies according to contraceptive type and individual patient characteristics. Combined hormonal contraceptives generally confer a greater increase in venous thromboembolism risk than in arterial thrombosis [15]. Obesity further amplifies this risk, as obese hormonal contraceptive users demonstrate higher rates of venous thromboembolism compared with their non-obese counterparts. This interaction highlights the necessity of incorporating body mass index and other prothrombotic factors into clinical decision-making [14].

Comprehensive cardiometabolic profiling is therefore essential prior to initiating hormonal contraception. Hypertension, diabetes, obesity, and dyslipidemia represent significant risk factors that may be exacerbated by hormonal contraceptive use [12]. Moreover, estrogen-induced alterations in microvascular endothelial function can increase the risk of cardiovascular disease, particularly among women with pre-existing cardiometabolic conditions [16]. 

Organ-specific vulnerability further complicates contraceptive selection. Estrogen and progestins can affect multiple organ systems, including cardiac, hepatic, renal, and neurological structures. Estrogen-induced microvascular endothelial dysfunction has been associated with increased cardiovascular risk [16]. Hepatic metabolism plays a central role in contraceptive processing and may influence liver function, while potential renal and neurological effects remain areas requiring further investigation [12]. Recognition of these organ-specific considerations is critical in medically complex patients. Given these multifactorial influences, individualized risk categorization requires a multidisciplinary approach that integrates age, body mass index, family history, and the presence of comorbid conditions [13]. Effective risk stratification should systematically incorporate thromboembolic risk assessment, cardiometabolic profiling, and evaluation of organ-specific vulnerabilities to guide contraceptive choice and ongoing management [12,14].

Comparative overview of contraceptive methods

Combined hormonal contraceptives typically contain both estrogen and progestin, which act synergistically to inhibit ovulation and induce changes in the endometrium that reduce the likelihood of implantation. Ethinyl estradiol is the most used estrogen component; however, alternatives such as estradiol valerate have been associated with a potentially lower risk of venous thromboembolism [17,18]. Despite their contraceptive efficacy, these agents exert systemic effects that influence hemostasis and vascular biology. Combined hormonal contraceptives can increase the risk of venous thromboembolism through alterations in clotting factors and endothelial function, with the magnitude of risk varying according to the specific estrogen and progestin formulation used [13]. Beyond contraception, these methods provide additional clinical benefits, including cycle regulation, improvement of acne, and relief of dysmenorrhea, contributing to their widespread use among young women, including those with conditions such as inflammatory bowel disease [19]. Nevertheless, their use is limited in certain populations. Women with a history of thromboembolism or established cardiovascular disease may have absolute or relative contraindications to combined hormonal contraceptives, making individualized risk assessment essential prior to initiation [9,20].

In contrast, progestin-only contraceptives are available in oral, injectable, and implantable formulations and are frequently selected for women who cannot safely use estrogen-containing methods. These methods generally demonstrate a lower thrombotic risk profile compared with combined hormonal contraceptives, rendering them more suitable for women with elevated cardiovascular risk [13]. However, their safety profile is not entirely uniform. Certain formulations, such as depot medroxyprogesterone acetate, have been associated with reductions in bone mineral density and changes in body weight, factors that warrant consideration in long-term use [21]. Owing to their comparatively minimal thrombotic risk, progestin-only methods are often recommended for women with cardiovascular comorbidities [9].

Intrauterine devices represent another important category of contraceptive options, particularly for women requiring highly effective and long-acting methods. The copper intrauterine device exerts its contraceptive effect by inducing a localized inflammatory response within the uterine cavity that is toxic to sperm and ova [19]. In contrast, levonorgestrel-releasing intrauterine systems provide contraception primarily through local progestin effects on the endometrium, thereby minimizing systemic hormone exposure [13]. This predominantly local mechanism makes levonorgestrel-releasing devices especially suitable for women who cannot use systemic hormonal methods. For this reason, intrauterine devices are frequently recommended in patients with contraindications to systemic hormones, as their localized action limits systemic metabolic and thrombotic effects [19].

Permanent and barrier methods provide additional alternatives within the contraceptive spectrum. Surgical sterilization, including tubal ligation, offers definitive contraception but requires careful evaluation in medically complex patients, as procedural risks may be amplified by underlying comorbidities [9]. Barrier methods, such as condoms and diaphragms, constitute non-hormonal options that avoid systemic effects altogether; however, their effectiveness is generally lower than that of hormonal or intrauterine methods. Although barrier methods are considered safe for women with contraindications to hormonal contraception, their reliance on consistent and correct use represents a significant limitation in typical clinical practice [22].

Contraception in cardiovascular and thromboembolic disease

In women with cardiovascular conditions, contraceptive selection should prioritize safety with respect to thromboembolic and vascular risk. Progestin-only methods are generally preferred in this population because they are associated with a lower risk of thromboembolic events compared to combined hormonal contraceptives. Options such as the levonorgestrel intrauterine system and low-dose progestin-only pills have not been shown to increase the risk of venous thromboembolism, making them suitable alternatives for women at elevated cardiovascular risk [13]. 

Non-hormonal methods also represent safe and effective alternatives for women with cardiovascular and thromboembolic disorders. Barrier methods, copper intrauterine devices, and sterilization procedures do not influence clotting factors or exacerbate cardiovascular risk, thereby avoiding the prothrombotic and hemodynamic effects associated with estrogen-containing formulations [13]. In particular, the copper intrauterine device offers highly effective, long-acting contraception without systemic hormonal exposure, which is advantageous in medically complex patients. In contrast, combined hormonal contraceptives are generally contraindicated in women with uncontrolled hypertension, ischemic heart disease, heart failure, or a history of thromboembolic events. Their use in these populations is associated with an increased risk of venous thromboembolism and stroke, necessitating careful avoidance in high-risk individuals [23]. Similarly, estrogen-containing methods should be avoided in women with migraine with aura because of the elevated risk of cerebrovascular events observed in this subgroup [24]. 

Given the complexity of these decisions, multidisciplinary management is essential. Collaborative care involving cardiologists, gynecologists, and primary care providers allows contraceptive counseling to be tailored to each woman’s cardiovascular risk profile and overall clinical status. Comprehensive patient education should address the specific risks and benefits associated with different contraceptive methods, as well as the importance of adherence to prescribed regimens, to reduce misconceptions and improve contraceptive uptake among women with cardiovascular disease [9]. Furthermore, regular monitoring is recommended for women using hormonal contraceptives, including periodic assessment of blood pressure and other cardiovascular risk factors, to facilitate early detection and management of potential complications [23].

Contraception in metabolic and endocrine disorders

The interaction between diabetes mellitus and hormonal contraception requires careful metabolic consideration. Combined hormonal contraceptives have been associated with worsening glucose tolerance and an increased risk of type 2 diabetes mellitus, particularly among women at a later fertile age [25]. These findings highlight the need for cautious use in women with established metabolic vulnerability. In contrast, postmenopausal hormone therapy has been shown to reduce glycated hemoglobin and fasting glucose levels in women with type 2 diabetes mellitus, suggesting a potential benefit for glycemic regulation [26]. Furthermore, the use of oral estrogens in menopausal hormone therapy is recommended in women with low cardiovascular disease risk, as it may contribute to improved glycemic control in type 2 diabetes mellitus [27]. Although these data derive from menopausal therapy rather than contraceptive use, they underscore the complex and context-dependent metabolic effects of estrogen exposure. In women with insulin resistance and metabolic syndrome, contraceptive selection should aim to avoid exacerbating underlying metabolic dysfunction. Non-estrogenic options are generally favored in this population to minimize potential deterioration in glucose homeostasis. Progestin-only contraceptives are considered safer alternatives for women with obesity and insulin resistance due to their comparatively lower impact on glucose metabolism [28]. 

Obesity further complicates contraceptive decision-making because of its independent association with venous thromboembolism. The use of combined hormonal contraceptives in obese women is associated with an increased risk of venous thromboembolism, necessitating careful method selection. In this context, progestin-only products are recommended to reduce the cardiovascular risks linked to estrogen-containing contraceptives [28]. 

Dyslipidemia represents another important cardiometabolic consideration. Hormonal contraceptives can influence lipid profiles, potentially increasing atherosclerotic risk in susceptible women. Therefore, contraceptive choice should be individualized based on the patient’s lipid profile and overall cardiovascular risk factors [29]. 

In women with polycystic ovary syndrome, metabolic and reproductive factors intersect. Insulin sensitizers such as metformin and thiazolidinediones have been shown to improve metabolic profiles and alleviate symptoms of polycystic ovary syndrome, providing an alternative or adjunct to hormonal contraceptives. Hormonal contraceptives may be used to regulate menstrual cycles in this population; however, their impact on insulin resistance should be monitored carefully [30]. Balancing cycle control with metabolic safety remains central in these patients.

Thyroid disorders also warrant individualized consideration. Hormonal contraceptives can affect thyroid hormone levels and metabolism, necessitating caution in women with underlying thyroid dysfunction. Individualized contraceptive counseling is therefore essential to address the specific endocrine needs and metabolic context of women with thyroid disorders [29].

Contraception in autoimmune, neurological, and psychiatric conditions

Women with systemic lupus erythematosus face a heightened risk of adverse pregnancy outcomes, and many of the medications used in its management are teratogenic, making effective contraception essential [31,32]. Given the prothrombotic milieu associated with systemic lupus erythematosus, particularly in patients with antiphospholipid antibodies, estrogen-containing contraceptives are prescribed less frequently in women at high risk for thrombosis, in accordance with recommendations to avoid estrogen exposure in this setting [31]. For women with antiphospholipid syndrome, progestin-only contraceptives, intrauterine devices, and subdermal implants are recommended because of their lower thrombotic risk profile [33]. 

In rheumatoid arthritis, gaps in contraceptive counseling have been documented. Contraception documentation is often lacking overall, although higher documentation rates are observed among women receiving teratogenic medications. While biologic therapies used in rheumatoid arthritis do not directly interact with contraceptive methods, effective contraception remains necessary because other medications used in disease management may carry teratogenic risks [31].

Among women with inflammatory bowel disease, estrogen-based contraceptives are commonly used, particularly in younger populations, despite recommendations to avoid them because of an increased risk of thromboembolism. Long-acting reversible contraceptives are recommended as safer and more effective alternatives; however, they remain underutilized, partly due to patient discomfort and concerns about cost [19]. These barriers highlight the need for improved counseling and access to recommended methods. Neurological conditions also introduce specific contraceptive considerations. Enzyme-inducing antiepileptic drugs, including carbamazepine and oxcarbazepine, can reduce the effectiveness of oral contraceptives, resulting in higher rates of contraceptive failure. Women receiving these medications are advised to consider non-oral contraceptive methods to avoid drug-drug interactions that compromise contraceptive efficacy [34]. Migraine status further influences contraceptive eligibility; women with migraine without aura can generally use estrogen-containing contraceptives, whereas those with aura should avoid them because of an increased risk of stroke [19].

Psychiatric comorbidities also require individualized consideration. In women with depression, contraceptive choice should account for mood stability, as hormonal fluctuations may influence mood disorders. In cases of significant mood concerns, non-hormonal methods or progestin-only options may be preferable [19]. Across these conditions, drug-drug interactions remain a central concern, particularly with enzyme-inducing medications that significantly increase the risk of contraceptive failure. Comprehensive counseling regarding potential interactions and alternative contraceptive strategies is therefore essential for women receiving medications that affect hormonal contraceptive efficacy [34].

Contraception in hepatic, renal, and oncologic conditions

Hormonal contraception in women with chronic liver disease requires careful evaluation because hepatic metabolism plays a central role in hormone processing. In patients with hepatic adenomas or chronic liver disease, estrogen-containing contraceptives are generally contraindicated, as they may promote adenoma growth and increase the risk of rupture, potentially leading to life-threatening hemorrhage. Given this risk, non-hormonal methods or progestin-only options may represent safer alternatives; however, individualized risk assessment remains essential to account for the severity of liver dysfunction and overall clinical status [35].

Cirrhosis further complicates contraceptive management because impaired hepatic function alters hormone metabolism and may affect the pharmacokinetics of hormonal contraceptives. These changes can influence both efficacy and safety, potentially necessitating adjustments in contraceptive selection and dosing. To avoid exacerbating liver dysfunction or unpredictable systemic exposure, non-hormonal methods such as copper intrauterine devices are often recommended in women with cirrhosis [36].

Women with chronic kidney disease also present unique reproductive health challenges, including menstrual irregularities and low rates of contraceptive use. Altered metabolism and clearance associated with chronic kidney disease may affect the effectiveness of hormonal contraceptives, further complicating method selection. In this setting, non-hormonal methods or low-dose hormonal options may be considered, although patient-specific factors and potential interactions with dialysis must be carefully evaluated [37]. 

In post-transplant patients, including those who have undergone liver or kidney transplantation, contraceptive planning must account for potential interactions between immunosuppressive medications and hormonal contraceptives. These interactions may influence drug levels and compromise graft stability. As a result, barrier methods or non-hormonal intrauterine devices are often preferred to avoid pharmacologic interactions and maintain stable graft function [38]. 

Oncologic conditions introduce additional complexity. Hormonal contraceptives are generally contraindicated in women with current or prior hormone-sensitive malignancies, such as breast cancer, because of concerns regarding cancer recurrence. Non-hormonal contraceptive methods are therefore recommended, and fertility preservation strategies should be discussed with patients undergoing cancer treatment. Chemotherapy itself increases thrombotic risk, which may be further exacerbated by estrogen-containing contraceptives; consequently, progestin-only or non-hormonal methods are considered safer alternatives during active treatment [34].

Fertility preservation remains a critical consideration for women with oncologic diagnoses who may receive therapies that compromise reproductive capacity. The timing of fertility preservation interventions should be carefully coordinated with cancer treatment plans to optimize both oncologic and reproductive outcomes [34]. Multidisciplinary collaboration among oncologists, reproductive specialists, and primary care providers is essential to support patient autonomy and ensure comprehensive, individualized care [3].

Clinical decision-making, medical eligibility criteria, and monitoring

The application of evidence-based eligibility classification systems plays a central role in guiding contraceptive selection in women with medical comorbidities. The US Medical Eligibility Criteria provides updated recommendations for contraceptive use in women with conditions such as chronic kidney disease, thrombophilia, and cardiovascular disorders, enabling clinicians to base their decisions on current evidence [29]. These guidelines categorize contraceptive methods into four distinct groups according to safety and suitability, thereby assisting providers in identifying absolute and relative contraindications for each method [39]. 

Within this classification system, absolute contraindications refer to clinical scenarios in which the use of a specific contraceptive method poses a significant health risk. An example includes the use of combined hormonal contraceptives in women with a history of thromboembolic disorders, where the potential for harm outweighs any contraceptive benefit [9]. In contrast, relative contraindications describe conditions in which risks may outweigh benefits but do not categorically preclude use. In such cases, careful clinical judgment and close monitoring are required, as in women with controlled hypertension [40]. 

Algorithm-based tools further enhance clinical decision-making by integrating individual health profiles, comorbidities, and contraindications into structured pathways that streamline contraceptive selection [39]. These tools support evidence-based practice while reducing variability in care. However, algorithmic guidance should be complemented by shared decision-making, which involves engaging patients in meaningful discussions about their contraceptive options, health status, lifestyle considerations, and reproductive goals [41]. Reproductive life planning encourages women to articulate their future fertility intentions, thereby aligning contraceptive choice with long-term personal objectives [42].

In high-risk patients, counseling strategies must be comprehensive and supportive. Effective counseling addresses misconceptions, clearly communicates the risks and benefits associated with each contraceptive method, and ensures that patients feel empowered in their decisions [9]. Enhanced counseling interventions have been associated with increased contraceptive uptake without a corresponding rise in sexually transmitted infections, underscoring their value in clinical practice [42].

Ongoing follow-up is equally important, particularly in women with medical comorbidities. Regular appointments allow for assessment of contraceptive effectiveness, identification of adverse effects, and timely intervention when complications arise [39]. Providers should remain vigilant in monitoring changes in clinical status and be prepared to adjust contraceptive methods as necessary [43]. In situations where a patient’s medical condition becomes unstable, a safe transition to an alternative contraceptive method is critical, ensuring that the new option is compatible with the patient’s current health status and reproductive goals [40].

## Conclusions

Hormonal contraceptive selection in women with medical comorbidities must be grounded in pathophysiological understanding and individualized risk assessment. Estrogen-containing methods increase thromboembolic and cardiovascular risk through effects on coagulation, blood pressure, and endothelial function, while progestin-only options generally present a safer profile, although not uniformly. Cardiometabolic status, autoimmune activity, organ dysfunction, obesity, and drug interactions must be systematically evaluated to minimize adverse outcomes.

Safe contraceptive management in high-risk populations requires the application of evidence-based eligibility criteria, multidisciplinary collaboration, and shared decision-making. Progestin-only and non-hormonal methods are often preferred in women with significant comorbidities, whereas combined hormonal contraceptives may be contraindicated. Ongoing counseling, monitoring, and timely adjustment of therapy are essential to ensure safety and alignment with reproductive goals.
